# Preliminary Study on the Skin Lightening Practice and Health Symptoms among Female Students in Malaysia

**DOI:** 10.1155/2015/591790

**Published:** 2015-11-26

**Authors:** Siti Zulaikha Rusmadi, Sharifah Norkhadijah Syed Ismail, Sarva Mangala Praveena

**Affiliations:** Department of Environmental and Occupational Health, Faculty of Medicine and Health Sciences, Universiti Putra Malaysia (UPM), 43400 Serdang, Selangor, Malaysia

## Abstract

Many cases of dermatologic complication were reported with the use of skin lightening products. This study assessed the skin lightening practice and health symptoms among female students. Self-administered questionnaire was distributed to 104 female students (56 undergraduates and 48 postgraduates) aged 24 ± 2 years in Universiti Putra Malaysia. A total of 60.6% (*N* = 63) of the female students used skin lightening products (61.9% of undergraduates and 38.1% of postgraduates). Reasonable price (*N* = 35, 55.6%) and ingredients (*N* = 29, 46%) were considered the most important factors in the product selection. Most respondents purchased the product from drugstores (*N* = 39, 61.9%). Twenty-two respondents (34.9%) in this study experienced skin problem from the products they used. Skin peeling (*N* = 13, 12.5%) and acne (*N* = 9, 8.7%) were the most frequent symptoms experienced. Most of the respondents have the perception that lighter skin provides high self-esteem (*N* = 56, 53.8%) and looks beautiful and healthier (*N* = 54, 51.9%). The use of skin lightening products is common among female students in this study and some of these products can cause skin problems such as skin peeling, acne, and itching.

## 1. Introduction

Throughout Asia, associating white skin to wealth and desirability is not new. The belief and practice to have lighter skin has been rooted from ancient times. For instance, Chinese myth believes that pearls can lighten one's complexion by taking a small amount of pearl powder together with hot water every day. In West Country, aristocrats and rich people in the seventeenth and eighteenth centuries kept their skin white by applying lead oxide powder to their faces to differentiate themselves from the working masses [1]. The colonial legacy in South Asia is said to be one of the contributory factors for the belief that white is powerful and white is beautiful as normally the white race was the ruler and the dark natives were the ruled [2].

The preference to have white skin has driven the skin lightening industry. This phenomenon was reflected in the domination of skin lightening products in Asian skincare market with 60 percent of sales [1]. Skin lightening products are readily available from major cosmetics companies, from local convenience stores, and widely over the internet. These types of products are marketed as skin-evening creams, skin lighteners, skin brighteners, skin whiteners, skin toners, fading creams, or fairness creams [3].

Although both men and women engage in the skin lightening practice, of various sorts, women generally have higher rates of practice than men. This situation can be seen in Santo Domingo, as early as the sixteenth century; the Indian women used painful processes to bleach their skin, trying to become more attractive to colonizers [4]. In a study of 450 Nigerians who confessed the use of lightening creams, 73.3% were women and 27.6% were men [5]. There is also a case of a middle-aged Nigerian lady who was brought home to die from a Western European country because of chronic use of skin lightening creams [5]. Additionally, a study on Mali stated that women, specifically students (45%), frequently experienced complications related to lightening agents [6]. Aside from how gender plays important role in practicing skin lightening, range of age is also an important factor. A study done by Hamed et al. shows that the majority of samples focus on female age ranging between 20 and 30 years (50.3%) [7]. Another study by Askari et al. shows that 72.1% of respondents were within this age [8]. Study by Adebajo also shows that 51.6% of the respondents were aged between 20 and 29 years [9]. Ravichandran also stated that plenty of cases reported of side effects caused by lightening cream were among women in the age group of 20 to 30 years [10].

There is a widespread practice of skin lightening in Malaysia. A Synovate regional survey in 2004 revealed that 61% of women in Malaysia believed they looked younger with a fair complexion [11]. Skin lightening product is a huge market in Malaysia. The sales of skin lightening products increase 100% every year for the past five years in Malaysia [12].

There is active involvement of Malaysian authority known as the National Pharmaceutical Control Bureau (NPCB) on monitoring the safety of cosmetic products including the skin lightening products. The NPCB role is to ensure the quality of notified cosmetic products in the Malaysian market and ensure compliance towards standards/specification set by the Ministry of Health, Malaysia [13]. For instance, some of the skin lightening products underwent a notification cancelation if the product was found to be adulterated with prohibited substances such as mercury or hydroquinone. Mercury is listed as a prohibited substance in the cosmetic products under the Guidelines for Control of Cosmetic Products in Malaysia [14], whilst hydroquinone is prohibited in cosmetic products and requires registration with the Drug Control Authority (DCA) and can only be used under the advice of a healthcare professional [15].

Study related to cosmetic practice in Malaysia is very limited, especially with regard to the practice and awareness of skin lightening. The aim of this study was to assess the practice of skin lightening and the attitude, knowledge, and perception among female students about the skin lightening practice. The information about health symptoms associated with the skin lightening practice among respondents will be collected. The findings of this study provide a baseline information on the current practice of skin lightening among female students in the country.

## 2. Methodologies

### 2.1. Questionnaire Development and Contents

Self-administered questionnaire was distributed to the respondents to study the behavior of skin lightening users and gather the information related to the practice of skin lightening, the knowledge, and also the perception about having white skin. The questionnaire consists of 5 sections in which section A determines the sociodemographic background of respondents, section B determines the skin lightening practice by the respondents, section C determines the consumer behavior and preference in skin lightening product selection, section D determines the health symptoms reported from the skin lightening products used, and section E determines the knowledge and perception of respondents towards skin lightening products. The reliability of the self-administered questionnaire was tested using Cronbach alpha.

### 2.2. Sampling Procedure

The study was carried out in Universiti Putra Malaysia, one of the public universities in Malaysia. The university is located in Serdang, 11 miles from the administrative capital city of Putrajaya. 104 respondents were recruited. A study was carried out from October to November 2014. While recruiting the respondents, the inclusion criteria implied were female students, aged from 20 to 30 and studying in the university on the period when the data is taken. Male students and female students aged under 20 were excluded from the study. As for the reason why the study only focuses on female students, it is because women generally have higher rates of skin lightening practice than men. As for the ethical clearance, the study protocol was approved by the Medical Research Ethical Committee of Universiti Putra Malaysia.

### 2.3. Data Analysis

Statistical analysis was performed using IBM SPSS Statistics 21.0 (SPSS Inc., Chicago, IL, USA). The Cronbach alpha test was run to determine the reliability of the self-administered questionnaire. A reliability coefficient of *α* > 0.70 indicates a high level of internal consistency. Descriptive analysis was used to describe the sociodemographic information and the skin lightening practice. Categorical data were summarized in frequency tables which include the number of samples and also percentage. The chi-square test was performed to determine the difference of mean variables between groups. Correlation test Spearman's Rho was performed to determine the relationship between variables in this study. The level of significance in this study was at *p* < 0.05.

## 3. Results and Discussion

This study involved 104 respondents that consist of 56 undergraduate students and 48 postgraduate students.  Table 1 illustrates the demographic profile of the respondents, including age, race, and monthly income or allowance. The majority of the respondents are the users of skin lightening products (*N* = 63, 60.6%). This value lies within the range reported in most of the previous study such as in Jordan (60.7%), Senegal (67.2%), and Lagos (72.4%) [7, 9, 16].

The majority of the respondents are Malays (84.6%, *N* = 88) and aged less than 25 years (*N* = 74, 71.2%). 87.5% (*N* = 49) of the undergraduate students received a monthly allowance less than 500 Malaysian Ringgit (MYR) while 50% (*N* = 24) of the postgraduate students received between MYR 1001 and MYR 2000. The age (*χ* ^2^ = 43.3, *p* < 0.001) and allowance received (*χ* ^2^ = 54.4, *p* < 0.001) were significantly different between groups.

Almost half of the female students in both groups (39 undergraduates and 24 postgraduate students) are currently using skin lightening products. The chi-square test indicates that there is a significant difference of skin lightening practice between undergraduate and postgraduate students in this study (*χ* ^2^ = 4.2, *p* = 0.04). The relationship between the education level and the skin lightening practice was significant at negative correlation (*r* = −0.20, *p* = 0.041).

Table 2 highlights the skin lightening practice among female students in this study. Based on the respondents who answer with* “Yes”* to the skin lightening practice, facial cleanser is the most common type of product used (*N* = 51, 81%) followed by facial moisturizer (*N* = 27, 39.7%). Other types of products are toner (*N* = 14, 22.2%), facial mask (*N* = 11, 17.5%), antiageing (*N* = 7, 11.1%), and sunblock and serum (*N* = 3, 4.8%).

Price (*N* = 35, 55.6%) and the ingredients (*N* = 29, 46%) are the two main factors that were considered the most in the skin lightening product selection. This was consistent with Dlova et al., where the price and brand were reported as the main factors in the product selection among two South African communities [17]. However, factor of brand in this study was less likely being considered by the respondent (*N* = 14, 22.2%). Other factors such as friend recommendations (*N* = 19, 30.2%) and the effectiveness (*N* = 15, 23.8%) were less likely being considered in this study. The advertisement was not the major factors influencing product selection by the respondent in this study (*N* = 17, 27%). However, in Hamed et al., advertisement was the major factor in the practice of skin lightening products by 77.4% of the female respondents in Jordan [7]. Interestingly, halal certification was also one of the factors considered in the product selection among a small group of students in this study (*N* = 6, 9.5%).

The international (35%) and local (33%) brand products are preferred among respondents. Undergraduate students prefer products less than MYR 30 (*N* = 22, 56.4%), while the postgraduate students prefer products between MYR 30 and MYR 60 (*N* = 11, 45.8%). The amount of income or allowance received per month is believed to be the reason on the pattern observed between these two groups, despite being at low significant correlation (*r* = 0.370, *p* = 0.003). Majority of the students in this study received a monthly income or allowance of less than MYR 500.

Majority of the respondents in this study checked the ingredient of the product before purchasing it (*N* = 33, 84.6% of the undergraduates and *N* = 20, 83.3% of the postgraduates). The reason of this practice was asked during the interview and all of them indicate that it is to ensure the safety of the products.

On the product satisfaction, a total of 55.6% (*N* = 35) of respondents were satisfied with the products they used where 21 of them are undergraduates and 14 postgraduates. Common reasons stated by the respondents who are not satisfied with their products were that the products do not give the expected result and they cause side effect to their skin. This finding was much lower compared to Dlova et al. and Hamed et al., where the satisfaction was recorded as 60% and 70.9%, respectively [7, 17].

The most common place to purchase the product in this study is in drugstore (*N* = 39, 61.9%), followed by department store (*N* = 19, 30.2%), convenience shop (*N* = 12, 19%), specialty beauty store (*N* = 12, 19%), and online (*N* = 8, 12.7%). A study in Jordan showed that drugstore (52.6%) and specialty beauty store (31.8%) are the common places to obtain skin lightening products [7].

Table 3 shows the skin problems experienced by respondents. 34.9% (*N* = 22) of respondents in this study had a skin problem from the application of skin lightening products where 14 of them are undergraduates and 8 postgraduates. Dlova et al. have reported 23% of Africans and 11% of Indians aged between 18 and 70 years had skin damage from the application of skin lightening products [17].

Out of 22 respondents who had a skin problem from lightening product application, the majority of them had skin peeling (*N* = 13, 59.1%), acne (*N* = 9, 40.9%), and itching (*N* = 8, 36.4%). Only 22.7% (*N* = 5) of respondents (3 postgraduates and 2 undergraduates) had consulted with the physician when they had a skin problem. Respondents were further asked if they underwent extensive treatment for their skin disease and only 4 respondents (80%) choose to do it to treat their problem. The reason stated by respondents on why they choose to undergo extensive treatment is because it was suggested by their physician and they also worried that the skin disorder might become more serious.

Chi-square test was run to indicate any significant difference on the perceived health symptoms experienced between undergraduate and postgraduate groups; nevertheless, no significant differences were identified.

The prevalence of disease in the population studied was the highest for skin peeling (*N* = 13, 12.5%), acne (*N* = 9, 8.7%), and itching (*N* = 8, 7.7%). There was no case of eczema reported. Mahé et al. report a study from Dakar, Senegal, where acne (29%), eczema (10%), skin irritation (3%), and itching (4%) were the common symptoms reported among women [18].

Table 4 shows the result of respondent knowledge and perception with regard to skin lightening practice. A majority of both undergraduates (*N* = 53, 94.6%) and postgraduate students (*N* = 44, 91.6%) are aware of the health effect caused by skin lightening products. This is consistent with a study in South Africa, where 89% of the respondents agree that skin lightening can produce adverse effect to their skin [17].

Most of the respondents in this study (*N* = 63, 60.6%) were able to name the ingredients that were banned from the skin lightening products such as mercury, hydroquinone, and tretinoin. Most of them knew about the banned ingredients from the internet and social networking site (SNS) (*N* = 92, 88.5%) and books, newspaper, and magazine (*N* = 60, 57.7%). The only small group of them (*N* = 44, 42.3%) got the information from friends. This result further was supported by the fact that 84.1% (*N* = 53) of those who use skin lightening products check the product's ingredient before purchase to ensure the safety.

Respondents in this study believe that having a lighter skin will provide higher self-esteem (*N* = 56, 53.8%) and looks beautiful and healthier (*N* = 54, 51.9%). They also have a perception that men consider women with lighter skin are more beautiful than a woman with dark skin (*N* = 34, 32.7%). Other perceptions about lighter skin were that lighter skin increases the chance of getting married (*N* = 7, 6.7%), lighter skin implies belonging to a higher social class (*N* = 6, 5.8%), and lighter skin helps in getting a better job (*N* = 4, 3.8%).

According to Robinson, the perception of having lighter or fairer skin is important to understand the motivations behind the practice of skin lightening among respondents [4]. A study by Askari et al. shows that the most common perception of respondents in Lahore is that men consider women with lighter skin to be more beautiful (82%), lighter skin tone increases woman's chance of getting married (70.5%), and lighter skin tone is more beautiful (59%). The least perception they have is that lighter skin tone implies that woman belongs to a higher social class (19.7%) [8]. Another study done by Hamed et al. shows that top three most common perceptions are that lighter skin tone increases woman's chance of getting married (63.4%), men consider woman with lighter skin to be more beautiful (62.6%), and lighter skin tone is more beautiful (62.3%). Same study also reported least perception on lighter skin tone implies that woman belongs to a higher social class with 25.2%.

The chi-square test was used to identify the significant difference of respondents' knowledge and perception between undergraduate and postgraduate students. There is no significant difference identified from the test.

## 4. Conclusion

In conclusion, female students aged from 20 to 30 are among those who practice skin lightening. Most of these students are well exposed to the information and have good knowledge on the safety of the products. They are able to name the ingredients that are banned from skin lightening products. However, despite knowing and being aware of the danger of skin lightening products, it does not stop them from using it as they believe that having a lighter skin tone is for their own self-satisfaction.

## Figures and Tables

**Table 1:** Sociodemographic background (*N* = 104).

Variables	Undergraduate^a^ *n* (%)	Postgraduate^b^ *n* (%)	Total^c^ *n* (%)
Age (years)^*∗∗*^			
20–24	55 (98.2)	19 (39.6)	74 (71.2)
25–30	1 (1.8)	29 (60.4)	30 (28.8)
Race			
Malay	47 (83.9)	41 (85.4)	88 (84.6)
Chinese	1 (1.8)	3 (6.3)	4 (3.8)
Indian	7 (12.5)	2 (4.2)	9 (8.7)
Others	1 (1.8)	2 (4.2)	3 (2.9)
Monthly income or allowance^*∗∗*^			
<MYR 500	49 (87.5)	10 (20.8)	59 (56.7)
MYR 500–MYR 1000	5 (8.9)	7 (14.6)	12 (11.5)
MYR 1001–MYR 2000	2 (3.6)	24 (50)	26 (25)
MYR 2001–MYR 3000	0 (0)	4 (8.3)	4 (3.8)
>MYR 3000	0 (0)	3 (6.3)	3 (2.8)
Use of skin lightening products^*∗*^			
Yes	39 (69.6)	24 (50)	63 (60.6)
No	17 (30.4)	24 (50)	41 (39.4)

*Note*. ^a^Total respondent (*N*) = 56, ^b^ *N* = 48, ^c^ *N* = 104, significant difference by chi-square test, ^*∗∗*^significant at *p* < 0.001, and ^*∗*^significant at *p* < 0.05.

**Table 2:** The skin lightening practice among female students (*N* = 63)^a^.

	Variables	Undergraduate (*N* = 39) *n* (%)	Postgraduate (*N* = 24) *n* (%)	Total (*N* = 63) *n* (%)
Types of products used	Facial cleanser	31 (79.5)	20 (83.3)	51 (81)
	Facial mask	5 (12.8)	6 (25)	11 (17.5)
	Facial moisturizer	16 (41)	11 (45.8)	27 (39.7)
	Antiaging	3 (7.7)	4 (16.7)	7 (11.1)
	Toner	9 (48.7)	5 (20.8)	14 (22.2)
	Others (sunblock, serum)	2 (5.1)	1 (4.2)	3 (4.8)

Factors considered during purchasing	Advertisement	10 (25.6)	7 (29.2)	17 (27)
	Friend recommendation	10 (25.6)	9 (37.5)	19 (30.2)
	Ingredient used	18 (46.2)	11 (45.8)	29 (46)
	Reasonable price	23 (59)	12 (50)	35 (55.6)
	Brand influence	7 (17.9)	7 (29.2)	14 (22.2)
	Effective within the short period of application	9 (23.1)	6 (25)	15 (23.8)
	Halal certification	3 (7.7)	3 (12.5)	6 (9.5)

Products preferred	Local	13 (33.3)	8 (33.3)	21 (33)
	International	12 (30.8)	10 (41.7)	22 (35)
	Neither of them	14 (35.9)	6 (25)	20 (32)

Price	<RM 30	22 (56.4)	6 (25)	28 (44.4)
	RM 30–RM 60	14 (35.9)	11 (45.8)	25 (39.7)
	>RM 60	3 (7.7)	7 (29.2)	10 (15.9)

Checking the ingredient	Yes	33 (84.6)	20 (83.3)	53 (84.1)
	No	6 (15.4)	4 (16.7)	10 (15.9)

Products satisfaction	Satisfied	21 (53.8)	14 (58.3)	35 (55.6)
	Not satisfied	18 (46.2)	10 (41.7)	28 (44.4)

Point of purchase	Department store	11 (28.2)	8 (33.3)	19 (30.2)
	Specialty beauty store	4 (10.3)	8 (33.3)	12 (9)
	Drugstore	24 (61.5)	15 (62.5)	39 (61.9)
	Online	3 (7.7)	5 (20.8)	8 (12.7)
	Convenience shop	8 (20.5)	4 (16.7)	12 (19)

Note: ^a^person who answers with *“Yes”* to skin lightening product use.

**Table 3:** Skin problems experienced by respondents (*N* = 63)^a^.

	Variables	Undergraduate (*N* = 39) *n* (%)	Postgraduate (*N* = 24) *n* (%)	Total *n* (%)
Skin problem caused by skin lightening products	Yes	14 (35.9)	8 (33.3)	22 (34.9)
	No	25 (64.1)	16 (66.7)	41 (65.1)

Type of skin problem^b^	Skin irritation	4 (28.6)	1 (12.5)	5 (22.7)
	Acne	7 (50)	2 (25)	9 (40.9)
	Eczema	—	—	—
	Itching	4 (28.6)	4 (50.0)	8 (36.4)
	Skin peeling	8 (57.1)	5 (62.5)	13 (59.1)
	Rashes	2 (14.3)	1 (12.5)	3 (13.6)
	Others	2 (14.3)	—	2 (9.1)

Referring to physician when facing skin problem^b^	Yes	2 (14.3)	3 (37.5)	5 (22.7)
	No	12 (85.7)	5 (62.5)	17 (77.3)

Undergoing extensive treatment^c^	Yes	2 (100)	2 (67)	4 (80)
	No	—	1 (33)	1 (20)

Note: ^a^person who answers with *“Yes”* to skin lightening product use; ^b^person who answers with *“Yes”* to skin problem cause by skin lightening products; ^c^person who answers with “*Yes*” to refer to physician when facing skin problem.

**Table 4:** Respondents knowledge and perception (*N* = 104).

Variables	Undergraduate (*N* = 56) *n* (%)	Postgraduate (*N* = 48) *n* (%)	Total (*N* = 104) *n* (%)
Knowing that skin lightening products might cause health effect			
Yes	53 (94.6)	44 (91.6)	97 (93.3)
No	3 (5.4)	4 (8.4)	7 (6.7)
Knowing ingredients banned from skin lightening products			
Yes	35 (62.5)	28 (58.3)	63 (60.6)
No	21 (37.5)	20 (41.7)	41 (39.4)
Way to obtain information about the safety of skin lightening products			
Television and radio	17 (30.4)	14 (29.2)	31 (29.8)
Internet and SNS	47 (83.9)	45 (93.8)	92 (88.5)
Book, newspaper, and magazine	31 (57.1)	29 (60.4)	60 (57.7)
Friend	23 (41.1)	21 (43.8)	44 (42.3)
Perception of having lighter skin			
Lighter skin is more beautiful and looks healthier	28 (50)	26 (54.1)	54 (51.9)
Lighter skin provides higher self-esteem	28 (50)	28 (58.3)	56 (53.8)
Lighter skin implies belonging to higher social class	3 (5.4)	3 (6.3)	6 (5.8)
Lighter skin helps in getting better job	2 (3.6)	2 (4.2)	4 (3.8)
Lighter skin increases chance of getting married	5 (8.9)	2 (4.2)	7 (6.7)
Men consider woman with lighter skin to be more beautiful	18 (32.1)	16 (33.3)	34 (32.7)
Do not have any perception	14 (25)	13 (27.1)	27 (26)
